# *Mycobacterium terramassiliense, Mycobacterium rhizamassiliense* and *Mycobacterium numidiamassiliense* sp. nov., three new *Mycobacterium simiae* complex species cultured from plant roots

**DOI:** 10.1038/s41598-018-27629-1

**Published:** 2018-06-18

**Authors:** A. Bouam, N. Armstrong, A. Levasseur, M. Drancourt

**Affiliations:** Aix-Marseille Univ, IRD, MEPHI, IHU Méditerranée-Infection, Marseille, France

## Abstract

Three slowly growing mycobacteria named strain AB308, strain AB215 and strain AB57 were isolated from the tomato plant roots. The 16S rRNA and *rpoB* gene sequence analyses suggested that each strain was representative of one hitherto unidentified slowly-growing *Mycobacterium* species of the *Mycobacterium simiae* complex. Genome sequencing indicated that each strain contained one chromosome of 6.015–6.029 Mbp. A total of 1,197, 1,239 and 1,175 proteins were found to be associated with virulence and 107, 76 and 82 proteins were associated with toxin/antitoxin systems for strains AB308, AB215 and AB57, respectively. The three genomes encode for secondary metabolites, with 38, 33 and 46 genes found to be associated with polyketide synthases/non-ribosomal peptide synthases and nine, seven and ten genes encoding for bacteriocins, respectively. The genome of strain AB308 encodes for one questionable prophage and three incomplete prophages, while only incomplete prophages were predicted in AB215 and AB57 genomes. Genetic and genomic data indicate that strains AB308, AB215 and AB57 are each representative of a new *Mycobacterium* species that we respectively named *Mycobacterium terramassiliense*, *Mycobacterium numidiamassiliense* and *Mycobacterium rhizamassiliense*.

## Introduction

Non-tuberculous mycobacteria are known to belong to some rhizospheres. An Illumina-based analysis of core actinobacteriome revealed that organisms of the genus *Mycobacterium* were the organisms the most commonly detected in roots of rice plants after organisms of the genera *Pseudonocardia* and *Dietzia*; whereas mycobacteria were less frequent in stem and absent in seeds^1^. Many *Mycobacterium* species have been detected in plants including *Mycobacterium poriferae*, *Mycobacterium intracellulare*, *Mycobacterium chubuense*, *Mycobacterium fortuitum*, *Mycobacterium neoaurum*, *Mycobacterium diernhoferi*, *Mycobacterium obuense* and *Mycobacterium cookie* which have all been detected in shoots of potted *Pogonatherum paniceum*; and *Mycobacterium mucogenicum* and *Mycobacterium ilatzarense* which have been detected in micro-propagated tissues of the same plant^2^. Also, *Mycobacterium phlei* isolated from the wheat rhizosphere^3^,exhibited beneficial effects on wheat growth under saline conditions^4^.

Here, using a drastic decontamination protocol and an improved culture medium for mycobacteria named MOD9^5^ we isolated three endophytic mycobacteria closely associated with the roots of tomato plants; and characterized them as representative of three new *Mycobacterium* species; expanding the repertoire of cultured rhizosphere mycobacteria; and characterizing features unique to rhizosphere mycobacteria.

## Results

**Figure 1:**
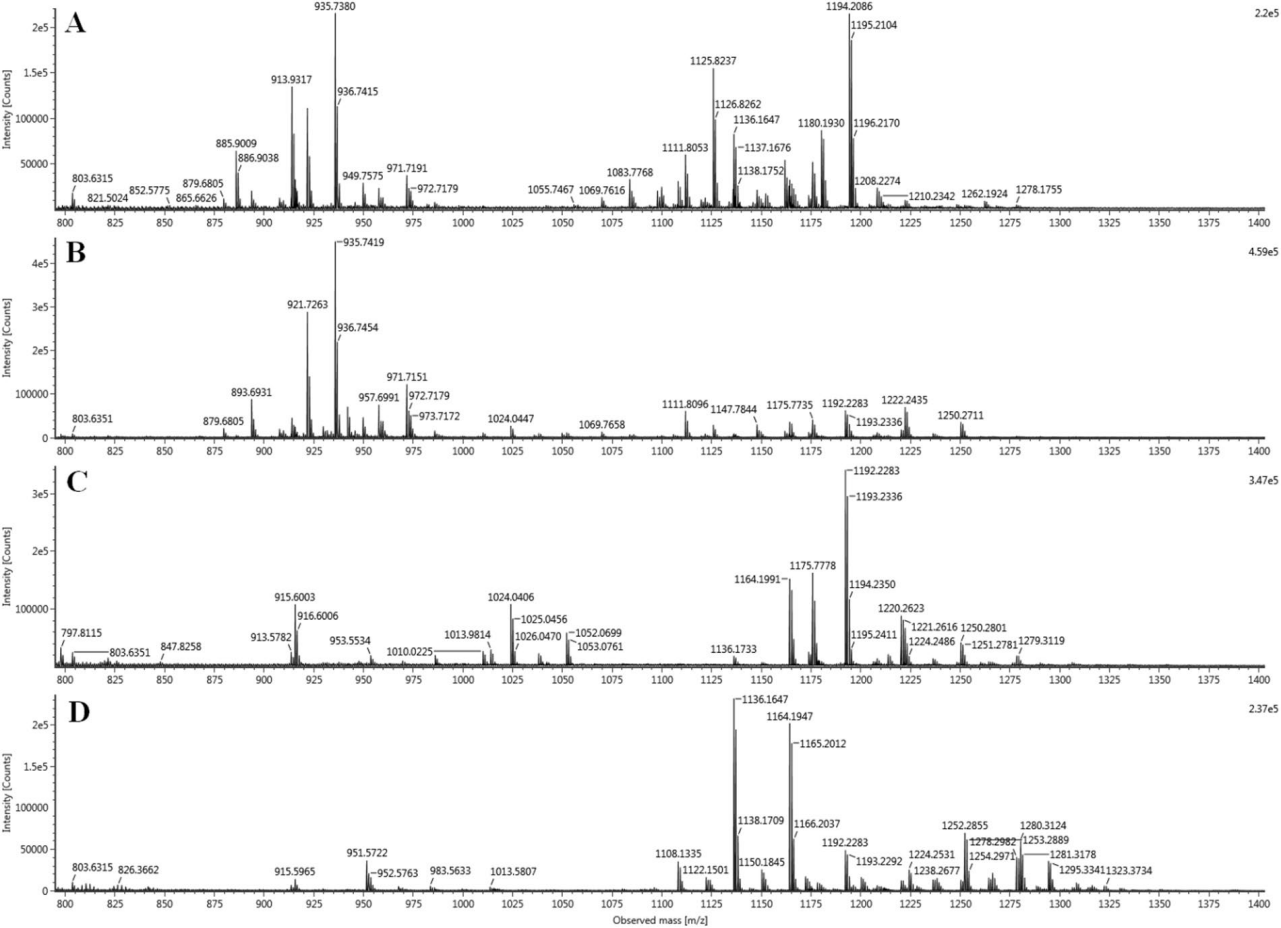
ESI-MS spectra of mycolic acids [M − H]^−^ ions. (**A**) *M*. *rhizamassiliense* AB57^T^, (**B**) *M*. *numidiamassiliense* AB215^T^, (**C**) *M*. *terramassiliense* AB308^T^, (**D**) *M*. *tuberculosis* H37Rv.

**Figure 2:**
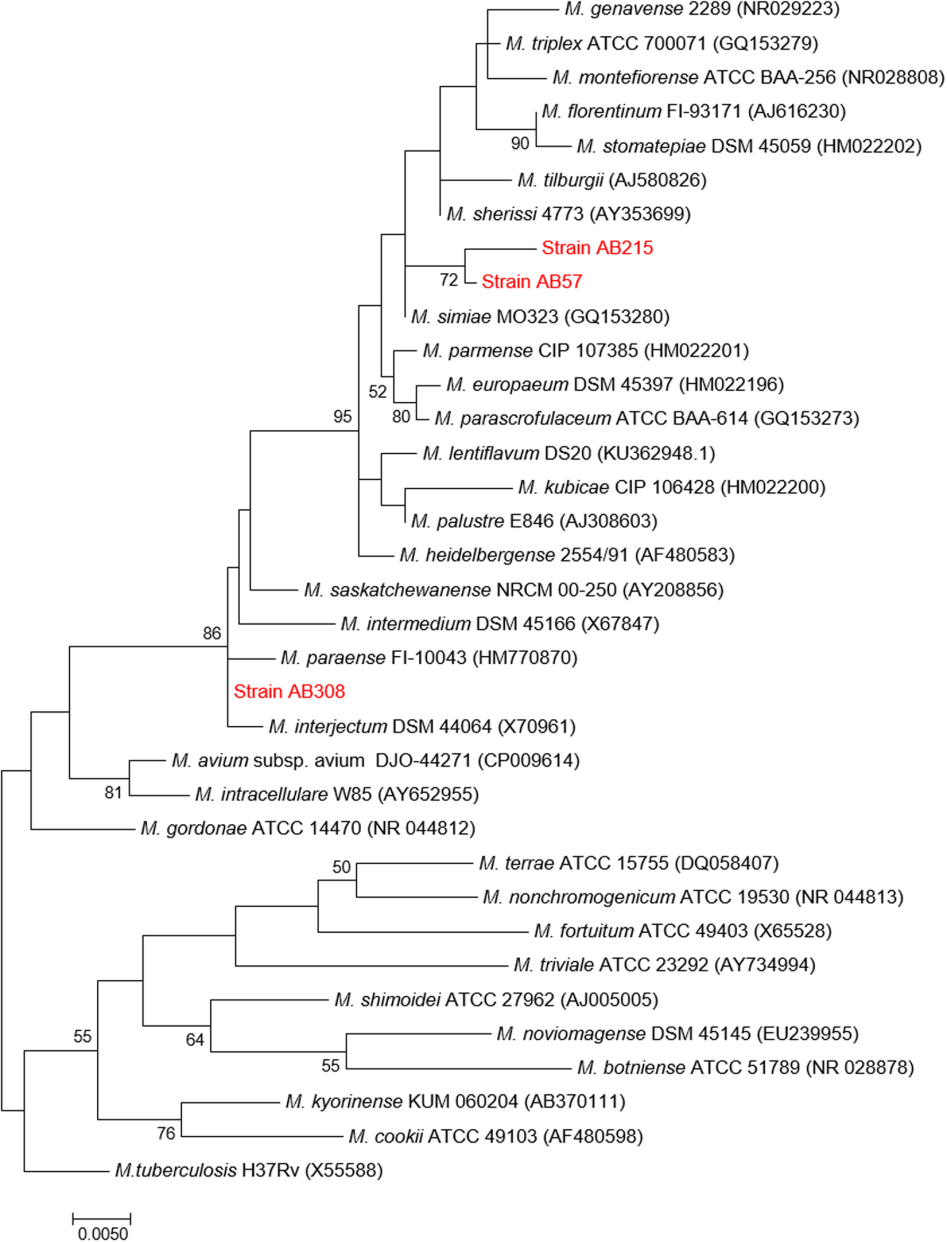
Phylogenetic tree based on the 16S rRNA gene sequence showing the phylogenetic position of AB308, AB57 and AB 215 strains within the *M*. *simiae* complex of mycobacteria including other mycobacteria species and *Mycobacterium tuberculosis* H37Rv as an out group. Sequences were aligned using CLUSTLE W implemented on MEGA7 33. The analysis includes 35 nucleotide sequences. Positions containing gaps and missing data were eliminated. There were a total of 1237 positions in the final dataset. Phylogenetic inferences obtained using the maximum likelihood method based on the Tamura and Nei model (bootstrapped 1000 times). Bootstrap values > 50% are given at nodes. Bar, 0.005 substitutions per nucleotide position.

After two-week incubation, 3/12 of secondary root-specimens yielded smooth and yellow colonies which were sub-cultured on Middlebrook 7H10 solid agar and designed as strain AB308, strain AB215 and strain AB57. Two of these strains were isolated from the roots of healthy plants and one from roots of diseased plant. The matrix-assisted laser desorption/ionization time of flight (MALDI-TOF-MS) peptide profile derived from strains AB308, AB215 and AB57 did not match any of the profiles entered in the Bruker database (version November, 2017); and differed from one strain to the two other ones. To better describe the three isolates, mycolic acids were extracted and subjected to electrospray-mass spectrometry analysis. Identified mycolic acids showed good mass accuracy (below 5 ppm error). The mass spectrometry analysis of strain *Mycobacterium tuberculosis* H37Rv (control) showed the previously described mycolic acid pattern^6,7^, including α- (C74–84), methoxy- (C80–90) and keto- (C80–89) forms (Table 1, Fig. 1). Strains AB57, AB215 and AB308 presented different mycolic acid patterns (Fig. 1). All the three strains showed α-, α′- and keto/epoxy/ω-1 mycolic acids. In addition, strain AB308 showed dicarboxy (or ω-carboxy) mycolic acids (Table 1). The 16S rRNA gene sequence of strain AB308 exhibited 99.8%, 99.7% and 99.3% highest sequence similarity with *Mycobacterium interjectum* ATCC 51457, *Mycobacterium paraense* FI-10043 and *Mycobacterium saskatchewanense* NRCM 00-250, respectively. The 16S rRNA gene sequence of strain AB57 showed 99.4%, 99.2% and 99% highest sequence similarity with *Mycobacterium simiae* MO323, *Mycobacterium montefiorense* ATCC BAA-256 and *Mycobacterium sherrisii* 4773, respectively. The 16S rRNA gene sequence of strain AB215 yielded 99.2% highest sequence similarity with *M*. *simiae* MO323, 98.9% with *M*. *montefiorense* strain ATCC BAA-256 and 98.8% with *M*. *sherrisii* 4773. Phylogenetic tree based on the16S rDNA gene sequence indicated that these three isolates are distinguishable from their closest species belonging to the *M*. *simiae* complex (Fig. 2). Further partial *rpo*B gene sequence of strain AB308 showed 97.9%, 97.4% and 90.6% highest sequence similarity with *M*. *interjectum* DSM 44064, *M*. *paraense* FI-10043 and *M*. *saskatchewanense* DSM 44616, respectively. As for strain AB57, it yielded highest sequence similarity of 95.3%, 94.9% and 94.3% with *Mycobacterium florentinum* DSM 44852, *Mycobacterium stomatepiae* DSM 45059 and *Mycobacterium genavense* FI-06288, respectively. As for strain AB215, highest sequence similarity scores were of 92.7%, 92% and 91.8 with *M*. *parmense* CIP 107385, *M*. *montefiorense* DSM 44602 and *Mycobacterium heidelbergense* DSM 44471, respectively. The partial *rpo*B gene sequencing was previously shown to be a useful marker to delineate new species in the genus *Mycobacterium*^8^. Accordingly, the results here reported led us to suspect that the three strains under study could be representative of three new species. Therefore, we aimed to further characterize each strain including genome sequencing.

**Table 1:** Identified mycolic acids for strains *M*. *rhizamassiliense* AB57^T^, *M*. *numidiamassiliense* AB215^T^, *M*. *terramassiliense* AB308^T^ and *Mycobacterium tuberculosis* H37Rv (control).

Mycolic acid subclass	Formula	Calculated [M − H]^−^	*strain* AB-57		*strain* AB-215		*strain* AB-308		*Mycobacterium tuberculosis H37RV*	
			Error (ppm)	%^a^	Error (ppm)	%^a^	Error (ppm)	%^a^	Error (ppm)	%^a^
α-	C_69_H_134_O_3_	1010.02602			4.5	2.7	−3.5	2.8		
	C_70_H_136_O_3_	1024.04167			2.9	6.0	−1.0	11.0		
	C_71_H_138_O_3_	1038.05732			3.7	2.3	−0.2	2.4		
	C_72_H_140_O_3_	1052.07297			1.0	2.7	−3.0	6.0		
	C_74_H_144_O_3_	1080.10427							1.3	0.3
	C_75_H_146_O_3_	1094.11992							4.8	0.3
	C_76_H_148_O_3_	1108.13557	2.0	4.4	−1.8	0.6			2.0	3.4
	C_77_H_150_O_3_	1122.15122	−1.0	0.6					−1.0	1.7
	C_78_H_152_O_3_	1136.16687	−1.9	11.7	1.9	2.2	−1.9	1.9	−1.9	23.2
	C_79_H_154_O_3_	1150.18252	1.7	0.5					−2.1	2.8
	C_80_H_156_O_3_	1164.19817	−3.0	4.6	0.8	7.9	0.8	15.4	−3.0	21.7
	C_81_H_158_O_3_	1178.21382							1.5	1.2
	C_82_H_160_O_3_	1192.22947			−1.0	13.7	−1.0	34.3	−1.0	5.9
	C_83_H_162_O_3_	1206.24512			−4.3	4.1	3.0	1.0		
	C_84_H_164_O_3_	1220.26077			1.2	1.5	1.2	9.0	1.2	1.6
	C_86_H_168_O_3_	1248.29207					−1.9	0.9		
α′-	C_60_H_118_O_3_	885.90082	0.1	9.1	−4.1	1.5				
	C_62_H_122_O_3_	913.93212	−0.4	19.0	−0.4	10.0	−0.4	0.6		
	C_64_H_126_O_3_	941.96342			−1.4	15.5				
keto/epoxy/ω−1	C_78_H_152_O_4_	1152.16179	−1.7	2.5						
	C_80_H_156_O_4_	1180.19309	−0.1	12.3					3.6	1.0
	C_81_H_158_O_4_	1194.20874	−0.1	30.3						
	C_82_H_160_O_4_	1208.22439	2.5	3.4	2.5	2.8			2.5	0.9
	C_83_H_162_O_4_	1222.24004	−0.8	1.5	2.9	15.3				
	C_84_H_164_O_4_	1236.25569			3.5	2.3	−3.7	1.5	3.5	1.4
	C_85_H_166_O_4_	1250.27134			−0.2	7.9	3.4	4.3	3.4	1.4
	C_86_H_168_O_4_	1264.28699					−2.2	1.0	−2.2	1.5
	C_87_H_170_O_4_	1278.30264			−3.5	1.0	3.6	2.0	0.1	4.0
	C_89_H_174_O_4_	1306.33394							2.6	0.5
methoxy-	C_80_H_158_O_4_	1182.20874							−4.2	0.4
	C_81_H_160_O_4_	1196.22439							−2.5	0.8
	C_82_H_162_O_4_	1210.24004							−4.8	0.7
	C_83_H_164_O_4_	1224.25569							−2.1	2.5
	C_84_H_166_O_4_	1238.27134							−3.0	1.7
	C_85_H_168_O_4_	1252.28699							−1.2	6.9
	C_86_H_170_O_4_	1266.30264							−5.0	2.2
	C_87_H_172_O_4_	1280.31829							−4.6	6.3
	C_88_H_174_O_4_	1294.33394							−1.2	3.6
	C_89_H_176_O_4_	1308.34959							0.7	1.3
	C_90_H_178_O_4_	1322.36524							0.2	0.7
dicarboxy -	C_65_H_126_O_5_	985.95325					−4.6	2.1		
	C_67_H_130_O_5_	1013.98455					−3.1	3.1		
	C_69_H_134_O_5_	1042.01585					−2.7	0.9		

(^a^Relative intensity was calculated from the sum of the identified mycolic acids).

### Characterization of strain AB308

**Figure 3:**
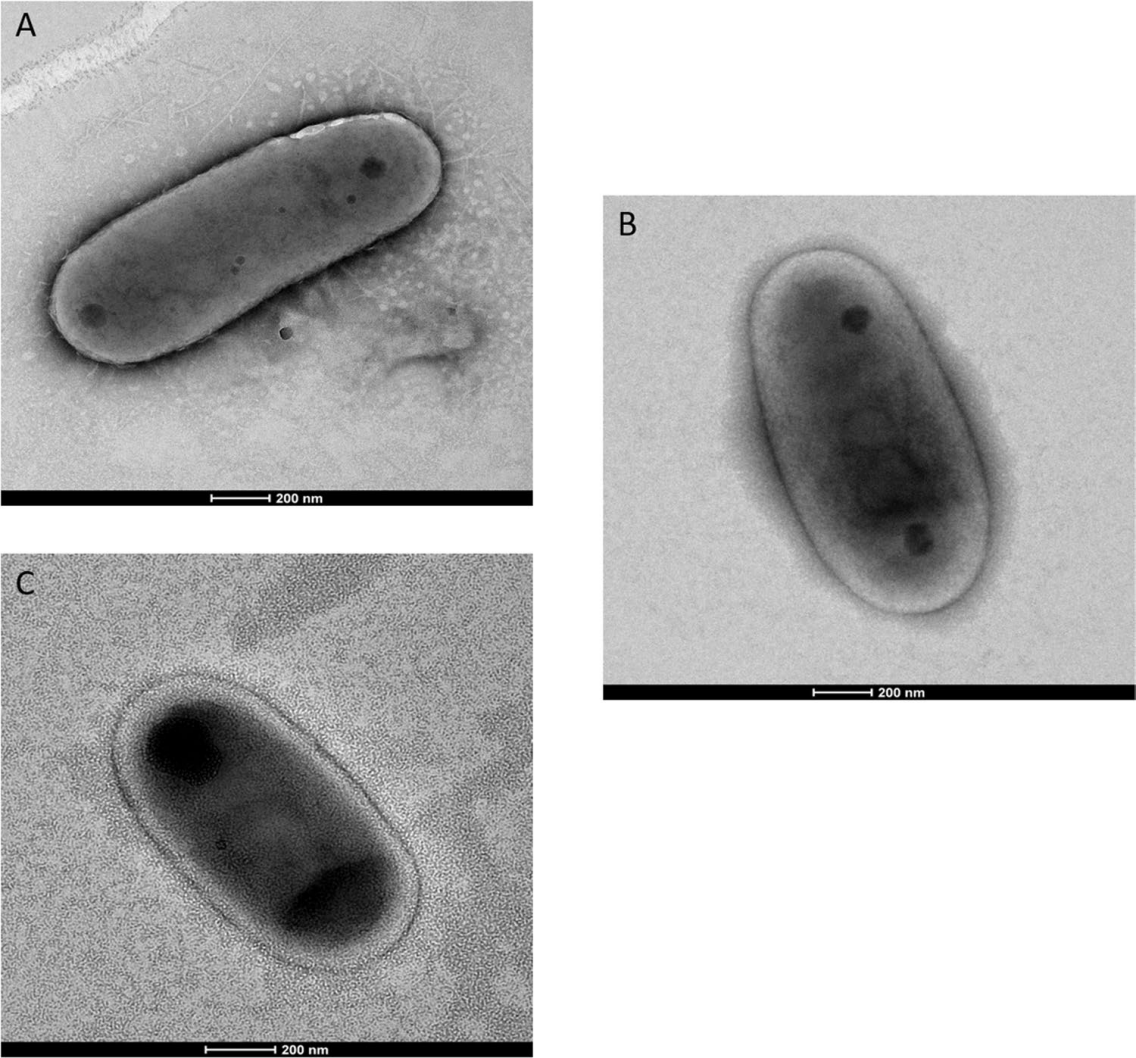
Transmission electron microscopy of *M*. *terramassiliense* strain AB308^T^ (**A**), *M*. *rhizamassiliense* strain AB57^T^ (**B**) and *M*. *numidiamassiliense* strain AB215^T^ (**C**). The scale bar represents 200 nm.

Strain AB308 exhibited scotochromogenic yellow, circular, smooth colonies on Middlebrook 7H10 medium within two weeks but culture was negative in 2%-salt Middlebrook 7H10 medium. Growth occurred in the range of 28–42 °C with an optimum growth at 37 °C. Strain AB308 exhibited Gram-positive and Ziehl-Neelsen stained red cells, which were non-motile and measured 0.58 ± 0.005 µm wide and 1.32 ± 0.09 µm long (Fig. 3A). The catalase test was positive and the oxidase test was negative. Strain AB308 tested positive for acid-phosphatase, esterase, leucine and valine arylamidase, lipase, naphtol-AS-BI-phosphohydrolase and tween 80 hydrolysis but tested negative for alkaline phosphatase, alpha and beta glucosidase, beta-glucuronisidase, esculin and gelatin hydrolysis, N-acetyl-beta-glucosaminidase, niacin production, nitrate reductase, pyrazinamidase and urease. This phenotypic profile differed from those of *M*. *interjectum* and *M*. *paraense*, the two closest organisms based on 16S rRNA gene sequence similarity: growth at 42 °C, negative urease and Tween 80 hydrolysis were distinguishing strain AB308 from *M*. *interjectum* and *M*. *paraense* (Table 2).

**Table 2:** Cultural and biochemical characteristics of strains AB308, AB57 and AB215 in comparison with the closely related Mycobacteria species.

Characteristics	AB308^T^	AB57^T^	AB215^T^	1	2	3	4
Growth at 42 °C	+	−	−	−	−	−	−
Growth <7 days	−	−	−	−	−	−	−
Catalase	+	+	+	+	+	+	+
Pigmentation	S	S	S	S	N	P	S
Nitrate Reduction	−	−	−	−	−	−	−
Niacin production	−	−	−	−	−	−	−
Urease	−	−	−	+	−	+	−
Tween 80 hydrolysis	+	−	−	−	−	−	−
Alkaline phosphatase	−	+	−	ND	ND	ND	ND
Lipase	+	+	−	ND	ND	ND	ND
Cystine arylamidase	−	+	−	ND	ND	ND	ND
Naphtol-AS-BI-phosphohydrolase	+	+	−	ND	ND	ND	ND
Tolerance 5% NaCl	−	−	−	ND	−	ND	ND

Species: 1, *M*. *interjectum*; 2, *M*. *montefiorense*; 3, *M*. *simiae*; 4, *M*. *paraense*; S, scotochromogenic; P, photochromogenic; N, nonphotochromogenic; ND, no data.

**Figure 4:**
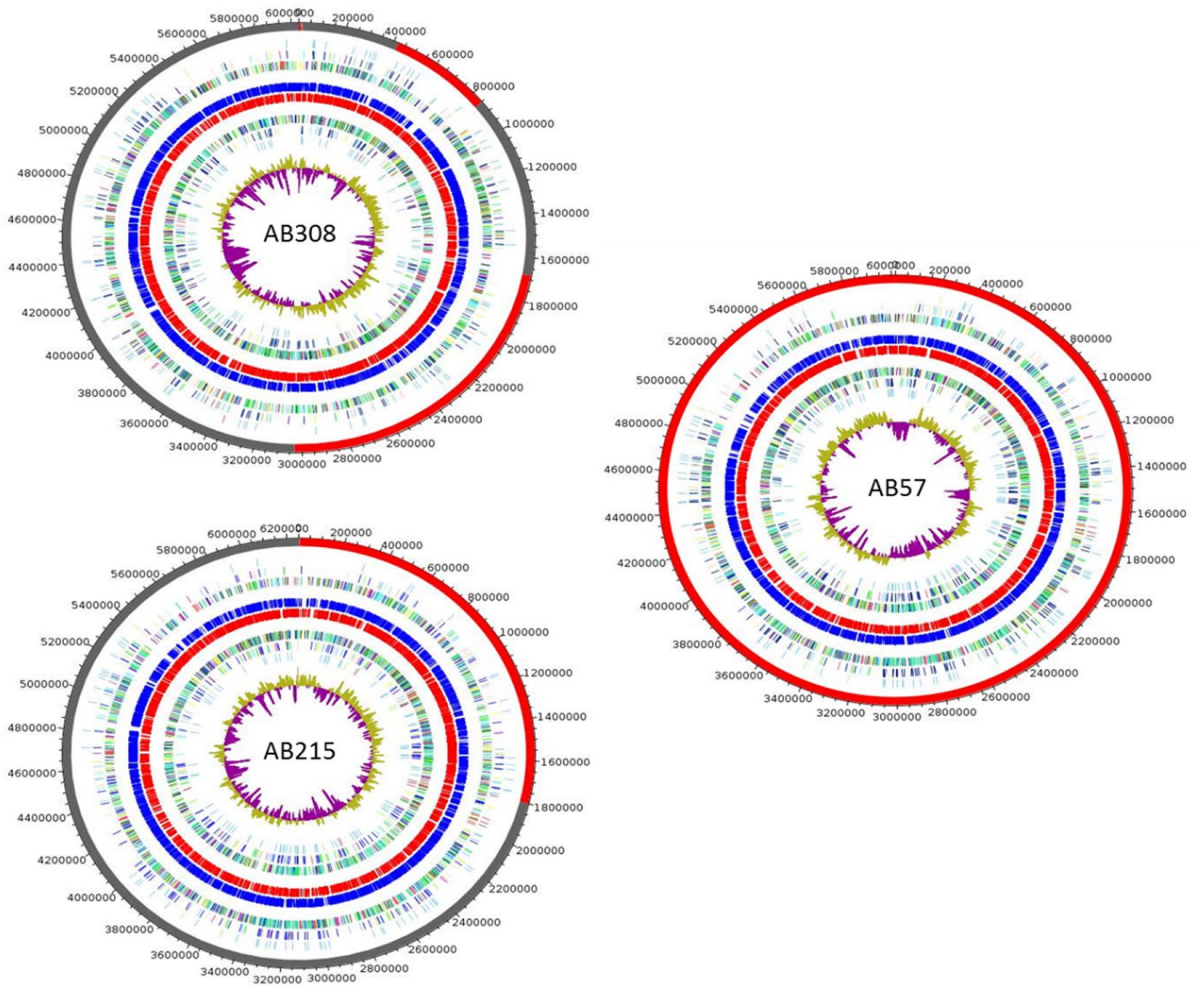
Graphical circular maps of the *M*. *terramassiliense* AB308^T^, *M*. *rhizamassiliense* AB57^T^ and *M*. *numidiamassiliense* AB215^T^ genomes. From outside to the center: Contigs (red/grey), COGs category of genes on forward strand (three circles), genes on forward strand (blue circle), genes on reverse strand (red circle), COGs category on reverse strand (three circles), GC content. COGs, Clusters of Orthologous Groups database.

**Figure 5:**
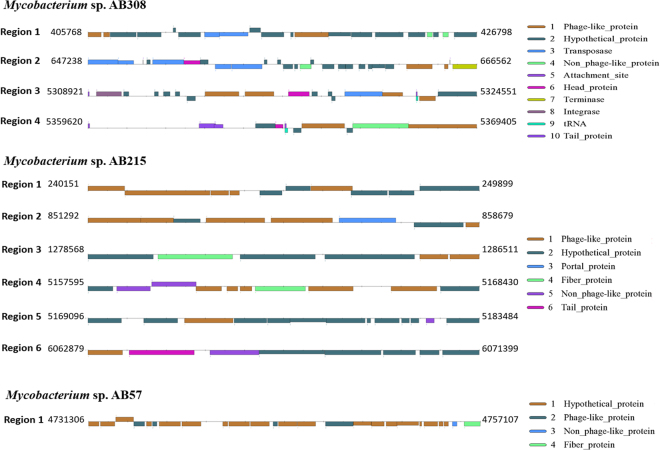
Genomic organization of *M*. *terramassiliense* AB308^T^, *M*. *numidiamassiliense* AB215^T^ and *M*. *rhizamassiliense* AB57^T^ prophages.

**Figure 6:**
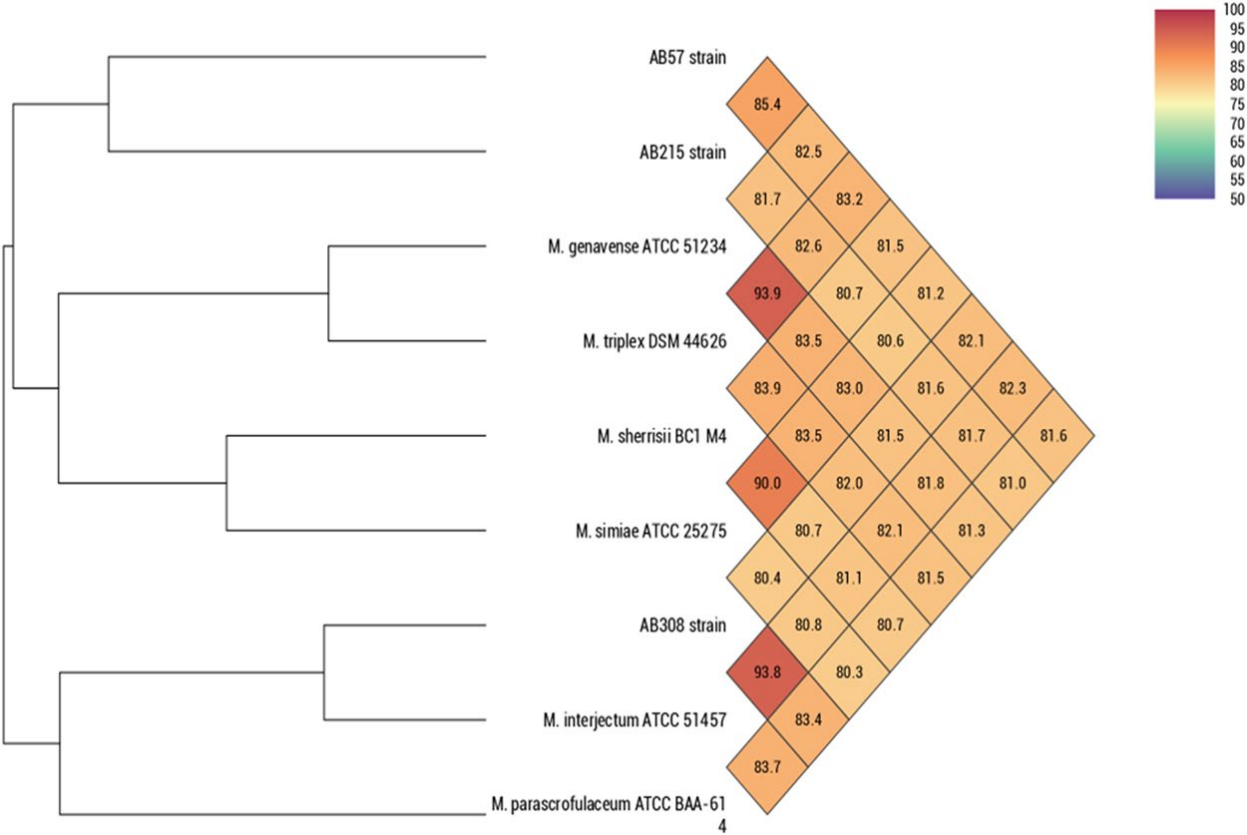
Heatmap fenerated with OrthoANI values of *M*. *terramassiliense* AB308^T^, *M*. *numidiamassiliense* AB215^T^ and *M*. *rhizamassiliense* AB57^T^ strains and other closest species of *M*. *simiae* complex calculated from the OAT software.

The draft genome sequence of strain AB308 was composed of 17 contigs assembled into 12 scaffolds without any extra-chromosomal replicons (Fig. 4). Analyzing the replication origin genome using Ori-Finder^9^ predicted one OriC region (647-bp) splited by the *dna*A gene (Supplementary File 2). The 647-bp predicted OriC region showed no homology sequence in DoriC database^10^. The 6,029,590-bp-long chromosome is smaller than those of *M*. *parascrofulaceum*, *M*. *triplex* and strain AB215 (6.56, 6.38 and 6.24 Mb respectively), but larger than those of strain AB57, *M*. *interjectum*, *M*. *simiae*, *M*. *sherrisii* and *M*. *genavense* (6.01, 5.84, 5.78, 5.68 and 4.93 Mb respectively). Its 68.39% GC content is smaller than those of *M*. *parascrofulaceum* (68.44%) but higher than those of *M*. *interjectum*, *M*. *genavense*, *M*. *sherrisii*, *M*. *triplex*, *M*. *simiae* and strains AB57 and AB215 (67.90, 67.22, 66.92, 66.92, 66.59, 66.16 and 65.85%, respectively). Its 5,678-gene content is smaller than those of *M*. *parascrofulaceum*, *M*. *triplex*, *M*. *interjectum* and strain AB215 (6,456, 5,988, 5,953 and 5,834, respectively) but larger than those of strain AB57, *M*. *simiae*, *M*. *genavense* and *M*. *sherrisii* (5,626, 5,533, 5,375 and 5,020, respectively). Of 5,730 genes predicted in the strain AB308 genome, 5,678 encode for proteins and 52 encode for RNAs including one complete ribosomal operon and 49 tRNAs. A total of 4,401 genes (77.51%) were assigned as putative function by COGs or by NR blast and 139 genes (2.45%) were identified as ORFans. The remaining 955 genes (16.82%) were annotated as hypothetical proteins. A total of 2,582 genes (45.47%) were found to be associated with the mobilome, including 217 phage proteins. Further genome analysis predicted one questionable prophage and three incomplete prophages (Fig. 5). COG analyses found (6.9%) genes coding for secondary metabolites biosynthesis, transport and catabolism, with 18 genes found to be associated with polyketide synthases and 20 genes with non-ribosomal peptide synthases. A total of 1,197 proteins were found to be associated with virulence, 107 proteins were associated with toxin/antitoxin systems (17 toxin, 26 antitoxin and 64 unidentified toxin/antitoxin proteins). Nine genes encode for bacteriocins while no gene was associated with the resistome. We identified many genes assigned to COG functional categories for transport and metabolism of lipids (9.67%), amino acid transport and metabolism (4.3%) and energy production and conversion (5.28%) (Table 3). Strain AB308 genome sequence exhibited respectively the highest average nucleotide identity and DNA-DNA hybridization (DDH) of 93.7%, 53.4% with *M*. *interjectum*, 83.4%, 26.4% with *M*. *parascrofulaceum* and 82.02%, 24.7% with strain AB57 (Fig. 6, Table 4). These phenotypic, genetic and genomic features indicated that strain AB308 was representative of a previously un-described species that we named *Mycobacterium terramassiliense* with strain AB308^T^ as the type strain.

**Table 3:** Number of genes associated in the *M*. *rhizamassiliense* AB57^T^, *M*. *numidiamassiliense* AB215^T^ and *M*. *terramassiliense* AB308^T^ genomes with the 25 general COG functional categories.

Code	Description	AB308		AB57		AB215	
		Value	% of total	Value	% of total	Value	% of total
[J]	Translation	180	3.17	179	3.18	176	3.02
[A]	RNA processing and modification	1	0.02	1	0.02	1	0.02
[K]	Transcription	172	3.03	159	2.83	160	2.74
[L]	Replication, recombination and repair	114	2.01	90	1.60	93	1.59
[B]	Chromatin structure and dynamics	0	0	0	0	0	0
[D]	Cell cycle control, mitosis and meiosis	30	0.53	26	0.46	35	0.60
[Y]	Nuclear structure	0	0	0	0	0	0
[V]	Defense mechanisms	115	2.02	143	2.54	129	2.21
[T]	Signal transduction mechanisms	100	1.76	85	1.51	105	1.80
[M]	Cell wall/membrane biogenesis	156	2.75	143	2.54	164	2.81
[N]	Cell motility	15	0.26	12	0.21	16	0.27
[Z]	Cytoskeleton	0	0	0	0	0	0
[W]	Extracellular structures	4	0.07	4	0.07	4	0.07
[U]	Intracellular trafficking and secretion	26	0.46	24	0.43	25	0.43
[O]	Posttanslational modification, protein turnover,chaperones	113	1.99	107	1.90	111	1.90
[X]	Mobilome: prophages, transposons	23	0.40	18	0.32	12	0.20
[C]	Energy production and conversion	300	5.28	284	5.05	291	4.99
[G]	Carbohydrate transport and metabolism	199	3.50	201	3.57	194	3.32
[E]	Amino acid transport and metabolism	244	4.30	223	3.96	232	3.98
[F]	Nucleotide transport and metabolism	73	1.29	69	1.22	69	1.18
[H]	Coenzyme transport and metabolism	230	4.05	212	3.77	212	3.63
[I]	Lipid transport and metabolism	549	9.67	677	12.03	645	11.06
[P]	Inorganic ion transport and metabolism	192	3.38	214	3.80	213	3.65
[Q]	Secondary metabolites biosynthesis, transport and catabolism	392	6.90	477	8.48	436	7.47
[R]	General function prediction only	514	9.05	545	9.69	536	9.19
[S]	Function unknown	183	3.22	161	2.86	166	2.84
_	Not in COGs	2429	42.77	2339	41.57	2534	43.43

The total percentage is based on the total number of protein coding genes in the annotated genomes.

**Table 4:** Comparison of AB308, AB57 and AB215 strains with related mycobacteria species using GGDC, formula 2 (DDH estimates based on identities/HSP length).

	AB57 strain	AB215 strain	AB308 strain
AB57 strain		29.20%	24.70%
AB215 strain	29.20%		24.50%
AB308 strain	24.70%	24.50%	
M. genavense ATCC 51234	25.20%	24.70%	24.30%
M. interjectum ATCC 51457	25.30%	24.80%	53.40%
M. parascrofulaceum ATCC BAA-614	24.40%	23.90%	26.40%
M. sherrisii BC1 M4	23.90%	23.20%	23.30%
M. simiae ATCC 25275	23.80%	23.30%	23.30%
M. triplex DSM 44626	26.10%	25.30%	24.60%

### Characterization of strain AB57

Strain AB57 exhibited scotochromogenic yellow, circular, smooth colonies on Middlebrook 7H10 medium within two weeks but culture was negative on 2%-salt Middlebrook 7H10 medium. Growth occurred at 28–37 °C with an optimum growth at 30 °C. Strain AB57 exhibited Gram-positive cells stained in red using the Ziehl-Neelsen staining. Cells were non-motile and measured 0.67 ± 0.01 µm wide and 1.32 ± 0.14 µm long (Fig. 3B). The catalase test was positive and the oxidase test was negative. Strain AB57 was positive for alkaline phosphatase, beta glucosidase, esterase, leucine and valine arylamidase, lipase and naphtol-AS-BI-phosphohydrolase but it was negative for alpha glucosidase, beta-glucuronisidase, esculin and gelatin hydrolysis, N-acetyl-beta-glucosaminidase, nitrate reductase, pyrazinamidase and urease. This phenotypic profile differed from those of *M*. *montefiorense* and *M*. *simiae*, the two closest organisms based on the 16S rRNA gene sequence similarity. Indeed, the type of pigmentation distinguished strain AB57 from *M*. *montefiorense* and *M*. *simiae* while negative urease test distinguished strain AB57 from *M*. *simiae* (Table 2).

The draft genome sequence of AB57 is composed of six contigs assembled into one 6,015,465-bp-long scaffold. Using Ori-Finder^9^, three OriC regions (488 bp, 793 bp and 110 bp) splited by the *dna*A gene were predicted (Supplementary File 2). The 793-bp predicted OriC region showed the highest homology sequence with *Mycobacterium indicus pranii* MTCC 9506, while the 488-bp and the 110-bp Oric regions showed no homology sequence in DoriC database^10^. The genome length of strain AB57 is smaller than those of *M*. *parascrofulaceum*, strain AB215, *M*. *triplex* and AB308 strain (6.56, 6.24, 6.38 and 6.02 Mb, respectively) but larger than those of *M*. *interjectum*, *M*. *simiae*, *M*. *sherrisii* and *M*. *genavense* (5.84, 5.78, 5.68 and 4.93 Mb, respectively). The 67.22% GC content of strain AB57 is smaller than those of *M*. *parascrofulaceum*, strain AB308 and *M*. *interjectum* (68.45, 68.38 and 67.90%, respectively), but larger than those of *M*. *genavense*, *M*. *sherrisii*, *M*. *triplex*, *M*. *simiae* and strain AB215 (66.92, 66.59, 66.16 and 65.85%, respectively). The 5,730-gene content of strain AB57 is smaller than those of *M*. *parascrofulaceum*, *M*. *triplex*, *M*. *interjectum* and strains AB215 and AB308 (6,456, 5,988, 5,953, 5,834 and 5,678, respectively) but larger than those of *M*. *simiae*, *M*. *genavense* and *M*. *sherrisii* (5,533, 5,375, 5,020, respectively). Of the 5,730 predicted genes, 5,626 were protein-coding genes and 53 were RNAs including a unique complete ribosomal operon and 50 tRNAs. A total of 4,427 genes (78.69%) were assigned as putative function by COGs or by NR blast and 127 genes (2.26%) were identified as ORFans. The remaining 991 genes (16.19%) were annotated as hypothetical proteins. A total of 2,588 genes (46%) were found to be associated with the mobilome, including 183 phage proteins. Further genome analysis predicted one incomplete prophage (Fig. 5). The genome of strain AB57 has the genetic potential to produce secondary metabolites, with 18 genes found to be associated with polyketide synthases and 28 genes with non-ribosomal peptide synthases. A total of 1,175 proteins were found to be associated with virulence, 82 proteins were associated with toxin/antitoxin systems (11 toxin, 15 antitoxin and 56 unidentified toxin/antitoxin proteins). Seven genes encoded for bacteriocins while no gene was associated with the resistome. We identified 12.03% genes assigned to COG functional categories for transport and metabolism of lipids, secondary metabolites biosynthesis, transport and catabolism (8.48%), amino acid transport and metabolism (3.96%) and energy production and conversion (5.05%) (Table 3). Strain AB57 genome sequence exhibited respectively the highest average nucleotide identity and DNA-DNA hybridization (DDH) of 85.4%, 29.2% with strain AB215, 83.1%, 26.1% with *M*. *triplex* and 82.4%, 25.2% with *M*. *genavense* (Fig. 6, Table 4). These phenotypic, genetic and genomic features indicated that strain AB57 was representative of a previously un-described species that we named *Mycobacterium rhizamassiliense* with strain AB57^T^ as the type strain.

### Characterization of strain AB215

Strain AB215 exhibited scotochromogenic yellow, circular, smooth colonies on Middlebrook 7H10 medium within two weeks but culture failed on 2%-salt Middlebrook 7H10 medium. Growth occurred at 28 °C–37 °C with an optimum growth at 30 °C. Strain AB215 exhibited Gram-positive, red Ziehl-Neelsen-stained cells which were non-motile and measured 0.65 ± 0.06 µm wide and 1.23 ± 0.18 µm long (Fig. 3C). The catalase test was positive and the oxidase test was negative. Strain AB215 was positive for beta-glucosidase, esterase, leucine and valine arylamidase but it was negative for acid and alkaline phosphatase, alpha glucosidase, beta-glucuronisidase, esculin and gelatin hydrolysis, N-acetyl-beta-glucosaminidase, nitrate reductase, pyrazinamidase and urease. This phenotypic profile differed from those of *M*. *montefiorense* and *M*. *simiae*, the two closest organisms based on the 16S rRNA gene sequence similarity: this type of pigmentation distinguished strain AB215 from *M*. *montefiorense* and *M*. *simiae* while negative urease test distinguished strain AB215 from *M*. *simiae* (Table 2). The negativity of alkaline phosphatase, lipase, cystine arylamidase and naphtol-AS-BI-phosphohydrolase further distinguished strain AB215 from strain AB57.

The 6,248,949-bp-long draft genome of strain AB215 is composed of four scaffolds (composed of four contigs). Three OriC regions (534 bp, 923 bp and 489 bp) splited by the *dna*A gene were predicted by Ori-Finder^9^ (Supplementary File 2). The 534-bp and the 489-bp predicted OriC regions showed no homology sequence in DoriC database^10^, while the 923-bp OriC region showed the highest homology sequence with *Mycobacterium indicus pranii* MTCC 9506. The genome length of strain AB215 is smaller than that of *M*. *parascrofulaceum* (6.56 Mb) but larger than those of *M*. *triplex*, strains AB308 and AB57, *M*. *interjectum*, *M*. *simiae*, *M*. *sherrisii* and *M*. *genavense* (6.38, 6.02, 6.01, 5.84, 5.78, 5.68 and 4.93 Mb, respectively). The 65.85% GC content of strain AB215 is lower than those of *M*. *parascrofulaceum*, strain AB308, *M*. *interjectum*, strain AB57, *M*. *genavense*, *M*. *sherrisii*, *M*. *triplex* and *M*. *simiae* (68.44, 68.38, 67.90, 67.22, 66.92, 66.92, 66.59 and 66.16%, respectively). The 5,888-gene content of strain is smaller than those of *M*. *parascrofulaceum*, *M*. *triplex* and *M*. *interjectum* (6,456, 5,988 and 5,953) but larger than those of strain AB308, strain AB57, *M*. *simiae*, *M*. *genavense* and *M*. *sherrisii* (5,678, 5,626, 5,533, 5,375 and 5,020, respectively). Of the 5,888 predicted genes, 5,834 were protein-coding genes and 53 were RNAs including one complete ribosomal operon and 50 tRNAs. A total of 4,484 genes (76.86%) were assigned as putative function (by COGs or by NR blast) and 198 genes (3.39%) were identified as ORFans. The remaining 986 genes (16.9%) were annotated as hypothetical proteins. A total of 2,620 genes (44.91%) were found to be associated with the mobilome, including 217 phage proteins. Further genome analysis predicted two incomplete prophage regions (Fig. 5). The genome of strain AB215 has the genetic potential to produce secondary metabolites, with 15 genes found to be associated with polyketide synthases and 18 genes with non-ribosomal peptide synthases. A total of 1,239 genes were found to be associated with virulence, 76 proteins were associated with toxin/antitoxin systems (eight toxin, 16 antitoxin and 52 unidentified toxin/antitoxin proteins). Ten genes encoded for bacteriocins while no gene was associated with the resistome. We identified 11.06% genes assigned to COG functional categories for transport and metabolism of lipids, secondary metabolites biosynthesis, transport and catabolism (7.47%), amino-acid transport and metabolism (3.98%) and energy production and conversion (4.99%) (Table 3). Strain AB215 exhibited respectively the highest average nucleotide identity and DNA-DNA hybridization (DDH) of 85.4%, 29.2% with strain AB57, 82.5%, 26.1% with *M*. *triplex* and 81.7%, 25.3% with *M*. *interjectum* (Fig. 6, Table 4).

These phenotypic, genetic and genomic features indicated that strain AB215 was representative of a previously un-described species that we named *Mycobacterium numidiamassiliense* with strain AB215^T^ as the type strain.

## Discussion

Investigating mycobacteria associated with tomato plant rhizosphere, we isolated and cultured three mycobacteria strains which exhibited phenotypic, genetic and genomic features indicative of three different, previously un-described species. Negative controls in culture experiments remained negative after a drastic decontamination of the root surface supported that indeed the three isolates are part of roots of the tomato plant. We showed the presence of mycobacteria only in the roots in agreement with an Illumina-based analysis of rice plants indicating that mycobacteria were abundant in roots, less abundant in stems and absent in seeds^1^. On the other hand, it was shown experimentally that *M*. *avium* can internalize in the roots of tomato plants and spread in its different tissues, namely stem, leaves and fruits^11^. We are here reporting on natural infection. Accordingly, the number of colony-forming units (CFUs) estimated in the roots was 60 CFU/g which is five logs lesser than the 10^6^ CFU/g detected in roots in the experimentally model^11^.

Our study is the third one reporting the culture of mycobacteria in plant roots after the isolation of a *Mycobacterium* related to *Mycobacterium sacrum* from root nodules of the wild legume *Sphaerophysa salsula*; and the isolation of *Mycobacterium frederiksbergens* from root nodules of the vegetable *Astragalus armatus*^12^. However, at least eleven isolates were recovered from plant rhizosphere, including one *Mycobacterium phlei* isolate^3^ and four *Mycobacterium poriferae* related isolates from soil rhizosphere of wheat plants^13^, four *M*. *gilvum* isolates from the rhizosphere of *Phragmites australis* plants^14^ and two *M*. *rhodesiae* related isolates from the rhizosphere of Arctic native plants^15^. Some of these rhizosphere mycobacteria can colonize plant roots and promote its growth (3), some others contribute to hydrocarbons biodegradation such as pyrene, benzo[*a*]pyrene and diesel in the rhizosphere^14,15^. These twelve *Mycobacterium* strains belong to the *Mycobacterium parafortuitum*^16,17^ and *Mycobacterium smegmatis* complexes^18^ whereas the three isolates here reported belong to the *M*. *simiae* complex, currently the largest *Mycobacterium* complex comprising 18 described species^19^.

Genome sequence-derived analyses of the Carbohydrate-Active Enzymes (Cazy) content in strains AB308^T^, AB215^T^ and AB57^T^ including Glycoside Hydrolases (GH), Glycoside Transferees (GT), Polysaccharide Lyases (PL) and Carbohydrate Esterases (CE) (Supplementary File 1) did not find statistically significant differences in comparison with mycobacteria isolated in the rhizosphere (*M*. *phlei*, *M*. *gilvum* and *M*. *rhodesiae*), mycobacteria detected in plants (*M*. *chubuense*, *M*. *fortuitum*, *M*. *neoaurum* and *M*. *intracellulare*) and with the specialized mammal pathogen *Mycobacterium tuberculosis*. Further genome analysis did not show the presence of structural nitrogen fixation genes (*nif*), namely *nif*H, *nif*D and *nif*K. However, the three genomes encode for *nif*U-like domains, the only common region between the *nif*U protein from nitrogen-fixing bacteria and rhodobacterial species^12^. The role of this protein is not fully elucidated but it has been proposed to be required for the full activation of nitrogenase^20^. Interestingly we found this protein only in strains AB308^T^, AB215^T^ and AB57^T^ but neither in the rhizosphere mycobacteria (*M*. *phlei*, *M*. *gilvum*, *M*. *rhodesiae* and *M*. *flavum*) nor in the mycobacteria detected in plants (*M*. *fortuitum*, *M*. *chubuense*, *M*. *neoaurum*, *M*. *intracellulare* and *M*. *ulcerans*). However we found *nif* U-like N terminal domain in the genome of all the above species including strains AB308^T^, AB215^T^ and AB57^T^; and in several unrelated mycobacteria including *Mycobacterium tuberculosis*. This observation suggests that the three new species here reported could be implicated in nitrogen fixation and have beneficial effect for the plant.

These three isolates being deposited into publicly available culture collections are now available for the community to further assess their characteristics and potential values.

### Description of *Mycobacterium terramassiliense* sp. nov

*Mycobacterium terramassiliense* (te.rra.mas.si.li.en’se. adj Massiliense of Massilia, the old Latin name for Marseille where the strain was isolated and terra the Latin name of soil.

Cells are Gram-stain-positive bacilli and are acid-alcohol-fast. Colonies are scotochromogenic yellow, circular, smooth and grow on 5% sheep blood agar, Lowenstein Jensen medium and on Middlebrook 7H10 agar at an optimal temperature of 37 °C after one-week incubation. *M*. *terramassiliense* exhibits positive catalase and Tween 80 hydrolysis and negative results for niacin production, nitrate reductase, oxidase test and urease. *M*. *terramassiliense* contains α, α′, keto/epoxy/ω-1 and dicarboxy/ω-carboxy mycolic acids and exhibits a 6,029,590-bp draft genome with 68.39% G + C content, 5,678 protein-coding genes and 52 predicted RNA genes.

The type strain AB308^T^ is deposited in the Collection de Souches de l’Unité des Rickettsies (CSUR P2565) and in the DSMZ-Deutsche Sammlung von Mikroorganismen und Zellkulturen GmbH (DSM 103275). Its genome is deposited in the GenBank under accession number (NZ_FTRV00000000).

### Description of *Mycobacterium rhizamassiliense* sp. nov

*Mycobacterium rhizamassiliense* (rhi.za.mas.si.li.en’se. adj Massiliense of Massilia, the old Latin name for Marseille where the strain was isolated and rhiza the Latin name of root.

Cells are Gram-stain-positive bacilli and are acid-alcohol-fast. Colonies are scotochromogenic orange yellow, circular, smooth and grow on 5% sheep blood agar, Lowenstein Jensen medium and on Middlebrook 7H10 agar at an optimal temperature of 30 °C after one-week incubation. *M*. *rhizamassiliense* exhibits positive catalase and negative results for niacin production, nitrate reductase, oxidase test, Tween 80 hydrolysis and urease. *M*. *rhizamassiliense* contains α, α′ and keto/epoxy/ω-1 mycolic acids and exhibits a 6,015,465-bp draft genome with 67.22% G + C content, 5,626 protein-coding genes and 53 predicted RNA genes.

The type strain AB57^T^ is deposited in the Collection de Souches de l’Unité des Rickettsies (CSUR P2566) and in the DSMZ-Deutsche Sammlung von Mikroorganismen und Zellkulturen GmbH (DSM 103273). Its genome is deposited in the GenBank under accession number (FUFA00000000).

### Description of *Mycobacterium numidiamassiliense* sp. nov

*Mycobacterium numidiamassiliense* (nu.mi.di.a.mas.si.li.en’se. adj Massiliense of Massilia, the old Latin name for Marseille and Numidia, an ancient Berber kingdom of the Numidians (202 BC–40 BC) where the inventor of this strain originates.

Cells are Gram-stain-positive bacilli and are acid-alcohol-fast. Colonies are scotochromogenic dark orange/yellow, circular, smooth and grow on 5% sheep blood agar, Lowenstein Jensen medium and on Middlebrook 7H10 agar at an optimal temperature of 30 °C after one-week incubation. *M*. *numidiamassiliense* exhibits positive catalase and negative results for niacin production, nitrate reductase, oxidase test, Tween 80 hydrolysis and urease. It contains α, α′ and keto/epoxy/ω-1 mycolic acids and exhibits a 6,248,949-bp draft genome with 65.85% G + C content, 5,834 protein-coding genes and 54 predicted RNA genes.

The type strain AB215^T^ is deposited in the Collection de Souches de l’Unité des Rickettsies (CSURP2567) and in the DSMZ-Deutsche Sammlung von Mikroorganismen und Zellkulturen GmbH (DSM 103274). Its genome is deposited in the GenBank under accession number (FUEZ00000000).

## Materials and Methods

A 500-mg quantity of secondary roots, main root, stem and leaves of twelve tomato plants were sterilized separately by immersion for 1 minute in a 30% bleach followed by another three rinses in distilled sterile water for 5 minutes, samples were then crushed using a sterile piston pellet in 2-mL sterile tubes-Eppendorf containing 1 mL of sterile phosphate buffered saline (PBS). The homogenates were than filtered through a 5 µM filter to remove the debris, 1 ml of each filtrate was then mixed with 3 mL chlorhexidin 1% in 15-mL tubes and shacked for 20 minutes and the remaining volume was then completed with PBS. The tubes were centrifuged at 4,000 g/min for 20 minutes, the supernatants were eliminated and 200 µL of PBS were added to the pellet, 100 µL were then cultured in MOD 9 medium^5^ and incubated at 30 °C.

### Morphological features

The colonies were sub-cultured on Middlebrook 7H10 solid agar (Becton Dickinson, Le Pont de Claix, France). Matrix-assisted laser desorption ionization-time of flight-mass spectrometry (MALDI-TOF-MS) profile did not match any of the profiles entered in the Bruker database (version December, 2015). Optical microscopy was performed using a Leica DM 2500 microscope (Leica, Wetzlar, Germany) after Gram staining and Ziehl-Neelsen staining and pictures were taken using a Nikon Digital Sight DS-U1 Camera System (Nikon, Tokyo, Japan). For electron microscopy, 10 µL of a bacterial suspension in PBS were deposited on a 400-mesh Formvar carbon-coated copper grid (Euromedex, Strasbourg, France) for 10 minutes after the glow discharge. The sample was contrasted using a 5% ammonium molybdate solution. Images were collected using a Tecnai G2 (FEI, Munich, Germany), operating at 200 keV and bacteria were measured using Image J software, version 1.

### Phenotypic characterization

Growth temperature (ranging from 28 °C to 45 °C) was observed after sub-culture on Middlebrook 7H10 solid agar (Becton Dickinson). Ability to grow on 0% to 10% NaCl was determined by supplementing the Middlebrook 7H10 solid medium with 0–10% NaCl. Carbon source utilization and enzyme activities were determined by inoculating a suspension of 6 McFarland on an Api Coryne strip and Api Zym (bioMérieux, Craponne, France) according to the manufacturer’s instructions with an incubation time of 24 hours. Tween 80 hydrolysis test was performed according to the method of Wayne^21^. Niacin accumulation was tested using BBL ™Taxo ™TB Niacin test strips (Becton Dickinson) following the manufacturer instructions. Pigment production in the dark and catalase activity were determined according to the standard procedures^22^.

### Extraction and analysis of mycolic acids

Mycolic acids were prepared as described previously^23^ with some modifications. At least 5 inoculation loops were collected from a culture plate and transferred into a glass tube containing 2 mL of potassium hydroxyde 9 M. Mycolic acids were hydrolysed at 100 °C during 2 hours. Three mL of 6 N hydrochloric acid were added. Free mycolic acids were then extracted with 2 mL of chloroform. The organic phase was collected and dried at 40 °C under a stream of nitrogen. Free mycolic acids were then dissolved in 100 µL of a methanol-chloroform mixture (50:50, v/v) and subjected to electrospray-mass spectrometry analysis after a 2,000 fold dilution in methanol. Samples were analyzed in the Sensitivity Negative ionization mode using a Vion IMS QTof high resolution mass spectrometer (Waters, Guyancourt, France). Samples were infused at a 10 µL/min flow, after fluidics wash with a chloroform/methanol solution (50:50) and monitored from 500 to 2,000 m/z during 2 minutes. Ionization parameters were set as follow: capillary voltage 2.5 kV, cone voltage 50 V, source and desolvation temperatures 120/650 °C. Mass calibration was performed automatically during analysis using a Leucine Enkephalin solution at 50 pg/µL (Lockmass 554.2620 m/z). Mass spectra were then combined between 800 and 1400 m/z and smoothed for subsequent data interpretation. Mycolic acids were described according to previously detailed structures^24,25^. Here, keto, epoxy and ω-1methoxy mycolic acid subclasses could not be distinguished because of their identical chemical formula.

### Protein profile analysis

A small part of a colony was picked on a Middlebrook 7H10 solid-medium using a sterile tip and applied directly on a ground-steel MALDI target plate. Then, one µL of a matrix solution (saturated α-cyano-4- hydroxycinnamic acid in 50% acetonitrile and 2.5% trifluoroacetic acid) (Bruker Daltonics) was used to over-lay the sample. After 5 minute-drying, the plate was loaded into the Microflex LT (Bruker Daltonics) mass spectrometer. Spectra were recorded following the parameters as previously described^26^. All signals with resolution ≥400 were automatically acquired using AutoXecute acquisition control in flexControl software version 3.0 and the identifications were obtained by MALDI Biotyper software version 3.0 with the Mycobacteria Library v2.0 database (version December, 2015).

### Genome sequencing

Genomic DNA was sequenced on the MiSeq Technology (Illumina Inc, San Diego, CA, USA) with the 2 applications: paired end and mate pair. Both strategies were barcoded in order to be mixed respectively with 11 other genomic projects prepared according to the Nextera XT DNA sample prep kit (Illumina) and with 11 others projects according to the Nextera Mate Pair sample prep kit (Illumina). To prepare the paired end library, 1 ng of gDNA was fragmented and amplified by limited PCR (12 cycles), introducing dual-index barcodes and sequencing adapters. After purification on AMPure XP beads (Beckman Coulter Inc, Fullerton, CA, USA), the libraries were then normalized and pooled for sequencing on the MiSeq. Automated cluster generation and paired end sequencing with dual indexed 2x250-bp reads were performed in a 9-hours run.

Total i²nformation of 9.0 Gb was obtained from a 1,019 k/mm^2^ cluster density with a cluster passing quality control filters of 90.2% (17,374,744 passed filtered reads). Within this run, the index representation was 8.20% for AB215, 8.09% for AB57 and 6.38% for AB308. The number of end reads was 1,424,260 for AB215, 1,405,843 for AB57 and 1,108,717 for AB308. These reads were trimmed and filtered according to the read qualities.

The mate pair library was prepared with 1.5 µg of genomic DNA using the Nextera mate pair Illumina guide. The genomic DNA sample was simultaneously fragmented and tagged with a mate pair junction adapter. The profile of the fragmentation was validated on an Agilent 2100 BioAnalyzer (Agilent Technologies Inc, Santa Clara, CA, USA) with a DNA 7500 labchip. The optimal size of obtained fragments was 5.043 kb for AB215, 6.48 kb for AB57 and 4.757 kb for AB308. No size selection was performed and 544 ng, 600 ng and 618 ng of tagmented AB215, AB57 and AB308 fragments were circularized. The circularized DNA was mechanically sheared to small fragments with optima on a bi modal curve at 421 and 881 bp for AB215, 283 and 1954 bp for AB57 and 395 and 668 bp for AB308 on the Covaris device S2 in T6 tubes (Covaris, Woburn, MA, USA).The library profile was visualized on a High Sensitivity Bioanalyzer LabChip (Agilent Technologies Inc, Santa Clara, CA, USA) and the final concentration library was measured at 16.97 nmol/l for AB215, 3.91 nmol/l for AB57 and 8.05 nmol/l for AB308. The libraries were normalized at 2 nM, pooled with 11 other projects, denatured and diluted at 15 pM. Automated cluster generation and 2x250-bp sequencing run were performed in a 39-hours run. This library was loaded on two different flow cells. For each run, global information of 5.3 and 7.2 Gb were obtained respectively from a 559 and 765 K/mm^2^ cluster density with a cluster passing quality control filters of 96.3 and 94.7% (10,450,000 and 14,162,000 passed filter clusters for each sequencing run). Within these runs, the index representation was determined to 8.51 and 7.62% for AB215, to 5.87% for AB57 and to 9.35 and 7.13% for AB308. The 888,760 and 1,079,096 paired end reads of AB215, the 831,999-paired end reads of AB57 and the 977,551 and 1,009,490 paired end reads of AB308 were filtered according to the read qualities. The reads were assembled using the SPAdes software (http://bioinf.spbau.ru/spades)^27^ and the obtained contigs were combined by use of SSPACE^28^ assisted by manual finishing and GapFiller^29^.

### Genome annotation and comparison

RNAmmer^30^, ARAGORN^31^, Rfam^32^, PFAM^33^ and Infernal^34^ were used to predict non-coding genes and miscellaneous features, Prodigal^35^ was used to predict Coding DNA sequences (CDSs). Functional annotation was achieved using BLAST+^36^ and HMMER3^37^ against the UniProtKB database^38^. The origin of replication was predicted using OriFinder5,6 (http://tubic.tju.edu.cn/Ori-Finder/) and homology with other OriC regions was searched using blast algorithm in DoriC database7 (http://tubic.tju.edu.cn/doric/). Prophase prediction was achieved using PHAST software^39^. For genomes comparison, we used the following species: *M*. *genavense* ATCC 51234, *M*. *interjectum* ATCC 51457, *M*. *parascrofulaceum* ATCC BAA-614, *M*. *sherrisii* BC1 M4, *M*. *simiae* ATCC 25275, *M*. *triplex* DSM 44626. OrthoANI^40^ was used to calculate ANI and DDH values were calculated using the GGDC version 2.0 online tool^41^.

### Phylogenetic analysis

Phylogenetic analyses based on the 16S rRNA gene sequence were performed using MEGA7^42^ with the maximum likelihood method and complete deletion option, based on the Tamura-Nei model for nucleotide sequences. Initial trees for the heuristic search were obtained automatically by applying the neighbor-joining and BIONJ algorithms to a matrix of pairwise distances estimated using the maximum composite likelihood (MCL) method. Statistical support for internal branches of the trees was evaluated by bootstrapping with 1000 iterations.

### Availability of data and materials

The datasets generated and/or analyzed during the current study are available at GenBank under accession numbers: *Mycobacterium terramassiliense* (NZ_FTRV00000000), *Mycobacterium rhizamassiliense*, (FUFA00000000), *Mycobacterium numidiamassiliense* (FUEZ00000000).

## Electronic supplementary material

Supplementary Dataset 1

Supplementary Info 1
