# Supplementary material for: Mycobacterium terramassiliense, Mycobacterium rhizamassiliense and Mycobacterium numidiamassiliense sp. nov., three new Mycobacterium simiae complex species cultured from plant roots
Source: Sci Rep. 2018 Jun 18;8:9309. doi: 10.1038/s41598-018-27629-1 (PMC6006331; doi:10.1038/s41598-018-27629-1)
Supplement: Supplementary file 2 — Supplementary Info 1 [file 41598_2018_27629_MOESM2_ESM.pdf]

***Mycobacterium terramassiliense*, *Mycobacterium rhizamassiliense* and *Mycobacterium numidiamassiliense* sp. nov., three new *Mycobacterium simiae* complex species cultured from plant roots.**

A. Bouam[1], N. Armstrong[1], A. Levasseur[1] and M. Drancourt*[1]

Supplementary file 1

Details of OriC regions predicted in the genome of strains AB308, AB215 and AB57.

Mycobacterium terramassiliense :

Genome size	6029590 nt
Genome GC content	0.6839
DnaA box distribution	[DnaA box distribution]
OriC length	647 nt
OriC AT content	0.3184
The number of DnaA box	3
The location of oriC region	3483469..3484115 nt
The location of dnaA gene	-
The extremes of GC disparity	3431648 nt (minimum), 6022463 nt (maximum)
The extremes of AT disparity	5893509 nt (minimum), 3960 nt (maximum)
The extremes of RY disparity	3431573 nt (minimum), 6025419 nt (maximum)
The extremes of MK disparity	6022453 nt (minimum), 3387045 nt (maximum)
Note	Note that the E. coli perfect DnaA box (ttatccaca) was searched for with no more than two mismatches. [Gene list (zcurve1.02)]
Z-curves	[Figure1] [Figure2]
OriC Sequence	The DnaA boxes identified in the below sequence are capitalized and also marked in bold, if any.
	catggtcgtcgatatgcacggccgccgcagccgggacatcgtcgacaaggcgatcaccgc<br>gctgctgaacctcgaacaccgcggcgcccagggcgccgagccgcgcagcggtgacggcgc<br>cggcatcctgattcaggtgccggacgcgttcctgcgcgaggtcgtggatttcgaactgcc<br>cgctgcgggcagctacgccaccggtatcgcgttcttgccgcagtcgtcgaaggacgccgc<br>gaccgcgtgcgccgc**GGTGGAGAA**gatcgccgagtccgagggcctgcaggtcatcggctg<br>gcgcaacgtgcccacggacgactcgtcgctgggtgcgctgtcccgcgacgcgatgcccac<br>gttccggcaggtgttcatgacgggcgcctccgggatggcgctggagcgacgcgcctacgt<br>ggtgcgcaagcgcgccgagcacgagctgggcaccaagggcccgggccaggatggcccggg<br>tcgcgaaaccgtttacttcccaagcctttccggtcagacct**TCATCTACA**agggcatgct<br>gaccaccccgcagctcaaggcgttttaccttgacctgcaagacgatcggctgaccagcgc

	gctgggcatcgtgcactcccgc**TTCTCCACG**aacaccttcccgtcgt

Mycobacterium numidiamassiliense

The information of genome and oriC region

Genome size	6248949 nt
Genome GC content	0.6585
DnaA box distribution	[DnaA box distribution]
OriC length	534 nt
OriC AT content	0.3914
The number of DnaA box	6
The location of oriC region	1772264..1772797 nt
The location of dnaA gene	-
The extremes of GC disparity	1848671 nt (minimum), 5310514 nt (maximum)
The extremes of AT disparity	5327831 nt (minimum), 1897876 nt (maximum)
The extremes of RY disparity	1848674 nt (minimum), 5246343 nt (maximum)
The extremes of MK disparity	5328449 nt (minimum), 1857179 nt (maximum)
Note	Note that the E. coli perfect DnaA box (ttatccaca) was searched for with no more than two mismatches. [Gene list (zcurve1.02)]
Z-curves	[Figure1] [Figure2]
OriC Sequence	The DnaA boxes identified in the below sequence are capitalized and also marked in bold, if any.
	tagcgggtagctgaaagggccac**TGTTCATAA**cactgtgcatcgtg**TGTGCATAT**ccttc<br>gacacctgtgttgagtgggt**TGTGGAGAA**cc**TGTGGATTA**aaacgcggcgttgaagcatc<br>cgtgcagatacgcgcagtacaccactgcacatttgg**TGTGGAGAA**caattcgctcggcgt<br>gtcgggccaggttactgcgtcgggcgtgttggctt**TCACCCACA**ttttcgccgagttacg<br>agccggttaaggcgcccatgcaccccggtcaccgaaatagttcgccaggtccgccgcatc<br>cgcgcgtctacctagccggctctgagcgctcacagagcgccaaacgagttccggcggcca<br>ccctcaggggcggccgccggacaaagcaaacagtgtcgagttagctgtccgcgacgacgt<br>ccagcgtgaactccacgttgacctccgggtgcaggtgcaccgacaccgagtgcgcaccga<br>tggccttgatgtgcgccttgggcagccggacgatgcgcttgtccaggttcgggc

The information of genome and oriC region

Genome size	6248949 nt
Genome GC content	0.6585
DnaA box distribution	[DnaA box distribution]
OriC length	923 nt
OriC AT content	0.3792
The number of DnaA box	4
The location of oriC region	1846020..1846942 nt
The location of dnaA gene	-
The extremes of GC disparity	1848671 nt (minimum), 5310514 nt (maximum)
The extremes of AT disparity	5327831 nt (minimum), 1897876 nt (maximum)
The extremes of RY disparity	1848674 nt (minimum), 5246343 nt (maximum)
The extremes of MK disparity	5328449 nt (minimum), 1857179 nt (maximum)
Note	Note that the E. coli perfect DnaA box (ttatccaca) was searched for with no more than two mismatches. [Gene list (zcurve1.02)]
Z-curves	[Figure1] [Figure2]
OriC Sequence	The DnaA boxes identified in the below sequence are capitalized and also marked in bold, if any.
	aagcgcgccgcggcccttgcggcgccgaccggacacgatggcacgcccggcgcgggtacg<br>catgcgcagacggaagccgtgcacgcgggcacggcgccggttgttcggctgaaaggtccg<br>cttgcccttggccacggcattctcctcggtctgtcttgctcgaagctttcccagccatcc<br>ggccgcccgcgttttcaccgaatcgcggctcgatcgaaaagtggtcttgctactggccgg<br>cgcggttcccctgacatacctgggtcgcagccgcatcgccgactttcgggcgactgtatg<br>agggtactgatgaggattcgcctggtcaaacttggcctgccccaacggcaccagcggaac<br>tgcgaccccgaaccggcaccgcgccgccgaaaaacgaaaaatgccccacggcagcttaaa<br>gaagccgactggaatgcagcggaacggttggcagccgcacggaaaactgttagcttcggg<br>caatgccgtttcagaccccagcggcgcaagacaacgaagcaaggatggcggatcaactgg<br>ctgtctaggtgaacttctcgaggttccctccagcacggagatcgcgagccgtcgccggta<br>ggctgcggcccgtatacgatcacc**CTGTCCACA**cc**TGTGTATAA**cta**TGTGGACAG**atgc<br>ttcgtcgccaagcgtggttgtagctcaagcccagagtttgcgagaaccagggagatgcgt<br>cgttgaccgatgaccccggttcgggctttaccagcgtatggaatgcagtcgtttccgaac<br>tcaatggcgacgccagcgcggacggtctcaccaccaatcgaacggt**TCTGGACAA**cccgc<br>taacccctcagcaacgagcctggctgaatctcgttcagccactcaccatcgtcgaggggt<br>ttgccctgctgtctgtgccgagc

The information of genome and oriC region

Genome size	6248949 nt
Genome GC content	0.6585
DnaA box distribution	[DnaA box distribution]
OriC length	489 nt
OriC AT content	0.4376
The number of DnaA box	5
The location of oriC region	1947253..1947741 nt
The location of dnaA gene	-
The extremes of GC disparity	1848671 nt (minimum), 5310514 nt (maximum)
The extremes of AT disparity	5327831 nt (minimum), 1897876 nt (maximum)
The extremes of RY disparity	1848674 nt (minimum), 5246343 nt (maximum)
The extremes of MK disparity	5328449 nt (minimum), 1857179 nt (maximum)
Note	Note that the E. coli perfect DnaA box (ttatccaca) was searched for with no more than two mismatches. [Gene list (zcurve1.02)]
Z-curves	[Figure1] [Figure2]
OriC Sequence	The DnaA boxes identified in the below sequence are capitalized and also marked in bold, if any.
	agaagaaacaacttagagatctaagtatcagtattaaggcctgtgcattctggggacagc<br>ctggcttctgtgcagctaggacctcgatggggg**TGTGGATGA**cagcgcgcgtggctgtgt<br>gcacggtgttgagcggg**TGGGGATGA**atgacagatgtacacggcggcgcggt**TCATCCAC**<br>**G**gtggtttgcctgct**TTTTCCACA**gcgctgcccacaccgtcaggg**TGTGGCTGA**tgtgac<br>agccgtgcgagaagtttttttgaaaaaagtttgactcgtgagtttcaaatcagcgcttgga<br>gcgctggcggatgcgagtcgtgagttctttaacgtggtcgaacacctcacgtcgctcggc<br>catctcggacaagatctttcgttgcgcatacatgaccgtggtgtggtcgcggccgaatgc<br>ctgtccgatcttcggcagcgagaggtcagtgagttcgcggcacaggtacatcgcgatctg<br>ccgcgactg

Mycobacterium rhizamassiliense:

Genome size	6015465 nt
Genome GC content	0.6722
DnaA box distribution	[DnaA box distribution]
OriC length	488 nt
OriC AT content	0.4344
The number of DnaA box	4
The location of oriC region	1721223..1721710 nt
The location of dnaA gene	-
The extremes of GC disparity	1721273 nt (minimum), 4895128 nt (maximum)
The extremes of AT disparity	4894759 nt (minimum), 644303 nt (maximum)
The extremes of RY disparity	1719935 nt (minimum), 4506867 nt (maximum)
The extremes of MK disparity	4895128 nt (minimum), 1705512 nt (maximum)
Note	Note that the E. coli perfect DnaA box (ttatccaca) was searched for with no more than two mismatches. [Gene list (zcurve1.02)]
Z-curves	[Figure1] [Figure2]
OriC Sequence	The DnaA boxes identified in the below sequence are capitalized and also marked in bold, if any.
	agcgtcccttcacctacaaaatttcagtccagtggctagccgcgtcccccgctagcgtg<br>gggcccccgaaacggggagtcgaagaacaaccgtagatcgtccggggccatcttgaaagc<br>taatcgcgaaggccgacaaccgcagtgatcgagacgcttgtaagcccctggggacgaggc<br>gtccccaaacctgttcttcaaagaagaaacgacgaagagatctcagtaccagtattaagg<br>gctgtgcaagt**TGGGGAGAA**gtgcactcctgtgcagctaggggagcggcggggg**TGTGGA**<br>**TGA**cgcggtgcgcaactgtgtgtcccgtgtgggtcgac**TGGGGATGA**attacagatgtac<br>accgatggccgattactgcacgggggtttgcgtggt**TTTTGCACA**agcctgcgcacatag<br>ccagcgtgtggctggtgtgacagacgtgtgggaagtttttttgaaaaagtttgactcgtgg<br>ggtgcaaa

The information of genome and oriC region

Genome size	6015465 nt
Genome GC content	0.6722
DnaA box distribution	[DnaA box distribution]
OriC length	793 nt
OriC AT content	0.3670
The number of DnaA box	3
The location of oriC region	1723229..1724021 nt
The location of dnaA gene	-
The extremes of GC disparity	1721273 nt (minimum), 4895128 nt (maximum)
The extremes of AT disparity	4894759 nt (minimum), 644303 nt (maximum)
The extremes of RY disparity	1719935 nt (minimum), 4506867 nt (maximum)
The extremes of MK disparity	4895128 nt (minimum), 1705512 nt (maximum)
Note	Note that the E. coli perfect DnaA box (ttatccaca) was searched for with no more than two mismatches. [Gene list (zcurve1.02)]
Z-curves	[Figure1] [Figure2]
OriC Sequence	The DnaA boxes identified in the below sequence are capitalized and also marked in bold, if any.
	cgacgcatctccctggttctcgcaaactctgggctgagctacaacctcgctgtgacgaca<br>aagcaa**CTGTCCACA**tag**TTATACACA**gg**TGTGGACAG**gatgatcgtatacgggccgcaa<br>cctaccggcgacggctcgcgatctccgcgctggaggcaaccccgtggaggtcgcctagac<br>agctagtcgatccgccatcctcgcttcgttgtcatgcgccgttgcagtctgaaacggcat<br>tgcccgaagctaacagttttccgaccggctgccaacagttcggctgcattccaatcggct<br>tctctagcctccttgggcgcgtctttctctcggccgccggacgggaggccgagcggcctg<br>cctgagcacgggtattttcggtgacgagtggtagtcgcggcgttgcgccaggacaagttt<br>gaccaggcgaagtgtcatcagtaccctcatacagtcgcccgaaagtcggcgatacggctg<br>cgacccgagccatctgtgcggggaccgcgccggccagcagcagttagaccactttccgac<br>tgacccgcgggttgcgaaacgcgagcggccggatggcaatgcgagacacaccgaggagaa<br>tgccgtggccaagggcaagcggacctttcagccgaataaccggcgccgagcccgtgtgca<br>cggcttccgtctgcgcatgcgtacccgcgccgggcgtgccattgtgtcgggtcggcgtcg<br>caagggtcgccgcgcgctttctgcctgatccgcgccgacaggttcgtgggcggtgctttc<br>ggcacgcaaccgt

The information of genome and oriC region

Genome size	6015465 nt
Genome GC content	0.6722
DnaA box distribution	[DnaA box distribution]
OriC length	110 nt
OriC AT content	0.4818
The number of DnaA box	3
The location of oriC region	1743438..1743547 nt
The location of dnaA gene	-
The extremes of GC disparity	1721273 nt (minimum), 4895128 nt (maximum)
The extremes of AT disparity	4894759 nt (minimum), 644303 nt (maximum)
The extremes of RY disparity	1719935 nt (minimum), 4506867 nt (maximum)
The extremes of MK disparity	4895128 nt (minimum), 1705512 nt (maximum)
Note	Note that the E. coli perfect DnaA box (ttatccaca) was searched for with no more than two mismatches. [Gene list (zcurve1.02)]
Z-curves	[Figure1] [Figure2]
OriC Sequence	The DnaA boxes identified in the below sequence are capitalized and also marked in bold, if any.
	ccgcggtcgccgacaagctcagctaaaaggtctttgcggttgacatggtttttgtcgtt**TGTGGGTAA**accagcagcactccgcg**TGTGGATCTGTGAAAAA**cagctgaa
